# TCCA: An Inter-Layer Spatiotemporal Cross-Attention Network for IIoT Attack Traffic Detection

**DOI:** 10.3390/s26206648

**Published:** 2026-10-21

**Authors:** Xianghui Sun, Bingfeng Xu

**Affiliations:** College of Information Science and Technology & Artificial Intelligence, Nanjing Forestry University, Nanjing 210003, China; sunxianghui@njfu.edu.cn

**Keywords:** industrial internet of things, intrusion detection, spatiotemporal feature learning, cross-attention, temporal convolutional network

## Abstract

The rapid advancement of the Industrial Internet of Things (IIoT) has significantly improved operational efficiency while expanding the attack surface of critical industrial infrastructure. However, conventional intrusion detection systems (IDSs) often struggle to accurately identify diverse attack traffic, particularly minority-class and difficult-to-detect attacks. Existing intrusion detection systems predominantly rely on serial spatiotemporal architectures. These methods typically adopt unidirectional or late-fusion strategies, which limit the interaction between spatial and temporal features, while recurrent computation further constrains inference efficiency. To address these limitations, this paper proposes TCCA, an inter-layer spatiotemporal cross-attention network for IIoT intrusion detection. TCCA adopts a parallel dual-branch backbone that combines a Convolutional Neural Network (CNN) for capturing local spatial correlations with a Temporal Convolutional Network (TCN) for modeling long-range temporal dependencies, thereby avoiding sequential recurrent computation. More importantly, bidirectional cross-attention modules are integrated between intermediate layers to enable dynamic and fine-grained mutual guidance between spatial and temporal representations throughout the feature extraction process. Comprehensive evaluations on the CICIDS-2017 and CIC IIoT 2025 datasets demonstrate that TCCA achieves superior detection performance, particularly for elusive threats such as web attacks while supporting the potential for edge deployment.

## 1. Introduction

The rapid development of the Industrial Internet of Things (IIoT) has fostered pervasive connectivity among sensors, industrial controllers, edge nodes, and cloud platforms, significantly improving operational automation and efficiency [1]. However, this highly connected environment also substantially expands the attack surface of critical industrial infrastructure. Unlike traditional IT systems, IIoT environments require strict real-time responsiveness, continuous availability, and operational safety. Consequently, successful cyber intrusions can cause severe industrial disruptions, physical damage, and data breaches [2,3,4]. Meanwhile, the diversity of IIoT traffic and the imbalance between attack categories pose significant challenges to intrusion detection. In particular, minority-class attacks may be underrepresented in training data, while certain attack types exhibit patterns that are difficult to distinguish from legitimate traffic. These characteristics can limit the effectiveness of conventional signature-based detection systems, particularly when dealing with previously unseen or evolving attack behaviors [5,6]. Therefore, developing an intrusion detection model that can accurately identify diverse attack patterns, including minority-class and difficult-to-detect attacks, while maintaining low latency and high throughput remains an important security challenge.

To address these challenges, existing learning-based Intrusion Detection Systems (IDSs) generally follow two approaches: traditional machine learning [7] and deep learning [8]. Traditional methods, such as SVMs, Decision Trees, and Random Forests, rely heavily on handcrafted statistical features, which limits their adaptability to evolving attack patterns [9]. In contrast, deep learning models can automatically learn hierarchical representations from raw or preprocessed traffic streams [10]. Specifically, Convolutional Neural Networks (CNNs) are widely used to extract local spatial correlations [11], whereas Recurrent Neural Networks (RNNs) and Long Short-Term Memory (LSTM) networks are commonly used to model temporal dependencies [12,13]. More recently, Temporal Convolutional Networks (TCNs) have gained attention for capturing long-range temporal contexts through dilated convolutions while supporting parallel computation [14]. Nevertheless, single-architecture models tend to emphasize only one feature dimension and therefore cannot fully characterize the coupled spatiotemporal dynamics of complex IIoT attack traffic.

To address this limitation, hybrid architectures that combine spatial and temporal learning mechanisms have been developed. A representative paradigm is the serial CNN-LSTM architecture [15,16], which extracts spatial features before passing them to recurrent units. However, this rigid and unidirectional structure prevents temporal context from guiding spatial feature extraction and may cause subtle spatial information to be lost before temporal modeling. Moreover, the recurrent computation of LSTMs introduces substantial latency, which can hinder real-time detection. To support concurrent processing, parallel dual-branch models [17] extract spatial and temporal representations through separate branches and combine them at the output stage. Although this design avoids the sequential delay of serial architectures, conventional fusion strategies, such as concatenation or addition, are typically applied at the final decision layer. As a result, intermediate spatial and temporal representations remain largely isolated, leading to shallow feature interaction and limiting the ability to capture deeply coupled attack patterns.

These limitations reveal a critical research gap: existing hybrid methods struggle to achieve deep spatiotemporal feature coupling while maintaining the computational efficiency required for real-time IIoT edge deployment. To address this gap, this paper proposes TCCA, an inter-layer spatiotemporal cross-attention network for IIoT intrusion detection. TCCA employs a parallel dual-branch backbone consisting of a CNN branch for capturing local spatial correlations and a TCN branch for modeling long-range temporal dependencies. This parallel design avoids recurrent loops and alleviates the sequential processing bottleneck of LSTM-based networks. More importantly, TCCA integrates bidirectional cross-attention modules between intermediate layers to prevent the two branches from becoming isolated. Through these modules, intermediate spatial features dynamically identify informative temporal patterns, while temporal features simultaneously refine spatial representations. This layer-wise mutual guidance enables fine-grained spatiotemporal interaction throughout the feature extraction process rather than relying on late-stage fusion. Extensive experiments on the CICIDS-2017 and CIC IIoT 2025 datasets demonstrate that TCCA achieves strong detection performance across diverse attack categories while maintaining low inference latency and high throughput. The results also provide evidence of its potential for future deployment in resource-constrained IIoT environments.

The main contributions of this paper are summarized as follows:

(1) We designed a parallel dual-branch framework that combines CNN and TCN to simultaneously extract spatial correlations and long-range temporal dependencies. This architecture avoids sequential computation bottlenecks and provides an efficient foundation for parallel inference in high-throughput IIoT networks.
(2) We introduced bidirectional cross-attention modules between intermediate layers to enable dynamic and fine-grained interaction between spatial and temporal representations. This mechanism addresses the shallow-fusion limitation of existing parallel models and improves the representation of complex patterns associated with minority and difficult-to-detect attacks.
(3) We conducted extensive evaluations on the CICIDS-2017 and CIC IIoT 2025 benchmark datasets. The experimental results demonstrate that TCCA achieves strong detection performance, particularly for elusive threats such as web attacks, while supporting its potential for future deployment in resource-constrained edge environments.

The remainder of this paper is organized as follows. Section 2 reviews related work on learning-based intrusion detection. Section 3 presents the detailed architecture of TCCA. Section 4 describes the experimental setup and presents comparative evaluations, ablation studies, and computational efficiency analyses. Section 5 discusses practical deployment considerations and limitations. Finally, Section 6 concludes the paper and outlines future research directions.

## 2. Related Work

This section briefly reviews existing learning-based intrusion detection methods, examines their key architectural limitations, and highlights the technical innovations of TCCA.

### 2.1. General Network Intrusion Detection Methods

Early learning-based intrusion detection systems mainly relied on traditional machine-learning algorithms, often enhanced by metaheuristic optimization or cost-sensitive strategies. Representative approaches include GA/PSO/ACO-optimized SVMs [18] and cost-sensitive Random Forests [19]. Although these methods are computationally efficient on small datasets, they depend heavily on manual feature engineering and have limited ability to capture the high-dimensional and nonlinear relationships in modern network traffic. Deep-learning architectures reduce this dependence by automatically learning hierarchical representations. CNNs are effective in capturing local spatial correlations [20], whereas recurrent models, including RNNs, GRUs, and LSTMs, are commonly used to model temporal dependencies [21]. However, recurrent computation limits parallel processing and increases inference latency. TCNs address some of these limitations by expanding the temporal receptive field through causal and dilated convolutions while retaining parallel computation [22]. Nevertheless, single-architecture models mainly focus on either spatial or temporal information and therefore have limited ability to capture coupled spatiotemporal dynamics.

To model spatial and temporal characteristics jointly, hybrid spatiotemporal architectures have been developed along two main directions. In Sequential Hybrid Architectures, serial cascades, such as CNN-LSTM models and CNN–GRU models [15,23,24], first extract spatial features and then pass them to recurrent units for temporal modeling. This fixed processing order enforces unidirectional information flow, preventing temporal context from guiding spatial feature learning at earlier stages. The use of recurrent units also introduces computational dependencies and inference latency. In parallel hybrid architectures, parallel configurations [25] process spatial and temporal representations simultaneously through independent branches, thereby avoiding the sequential delays of serial architectures. However, existing parallel models mainly rely on late-fusion strategies, such as concatenation, addition, or attention applied to final representations. Consequently, intermediate spatial and temporal features remain largely isolated during feature extraction, limiting their ability to provide dynamic and mutual guidance.

### 2.2. IIoT-Oriented Intrusion Detection Methods

Intrusion detection in IIoT environments introduces additional operational requirements. Industrial traffic generated by heterogeneous controllers and specialized communication protocols exhibits complex and dynamic distributions [26]. IIoT applications require strict real-time execution, making high throughput and low inference latency essential. Moreover, sophisticated attacks, such as infiltration and web attacks, may constitute only a small fraction of total traffic. Consequently, overall classification accuracy alone may not adequately reflect detection performance, particularly when minority-class or difficult-to-detect attacks exhibit low recall [27].

To address these requirements, IIoT-oriented intrusion detection models have adopted lightweight ensemble [28] and hybrid architectures, such as the cascaded CNN-Transformer model LCT-IDS [29]. Although these approaches improve feature learning and computational efficiency, cascaded architectures rely on unidirectional feature transfer, while Transformer-based attention may introduce additional computational overhead. Other studies have explored recurrent and attention-based architectures. For example, BiGAT-ID combines BiGRU, LSTM, and multi-head attention to capture temporal dependencies and contextual features in medical and industrial IoT traffic [30]. Similarly, an ARN-based IDS models temporal patterns in industrial control traffic, with slightly higher computational overhead than GRU [31]. However, recurrent architectures introduce sequential processing dependencies that may limit inference efficiency. Beyond network-side intrusion detection, complementary studies focus on protecting the transmitted content. For instance, Lin et al. [32] identified vulnerabilities in a video cryptosystem and proposed security improvements. Such work addresses a different security layer and complements network-side intrusion detection. Table 1 compares representative IDS architectures and highlights TCCA’s use of inter-layer bidirectional cross-attention between parallel CNN and TCN branches.

In summary, existing IIoT intrusion detection methods face three main limitations: insufficient interaction between intermediate spatial and temporal features, computational overhead from recurrent-based architectures, and challenges in balancing detection accuracy with inference efficiency. To address these limitations, TCCA combines parallel CNN–TCN branches with inter-layer bidirectional cross-attention, enabling fine-grained spatiotemporal feature interaction without recurrent computation. Experiments on benchmark datasets demonstrate its effectiveness across diverse attack categories, including minority-class and difficult-to-detect attacks, while achieving low inference latency and high throughput. These results highlight its potential for efficient IIoT intrusion detection.

## 3. Architecture of the TCCA Model

This section presents the detailed architecture of the proposed inter-layer spatiotemporal cross-attention network (TCCA). We first describe the input sequence construction and overall processing pipeline, followed by the parallel spatiotemporal feature extraction branches. We then introduce the inter-layer bidirectional cross-attention mechanism and the feature fusion strategy. Finally, we summarize the end-to-end processing procedure and analyze the computational complexity of TCCA.

### 3.1. Overall Architecture

**Figure 1:**
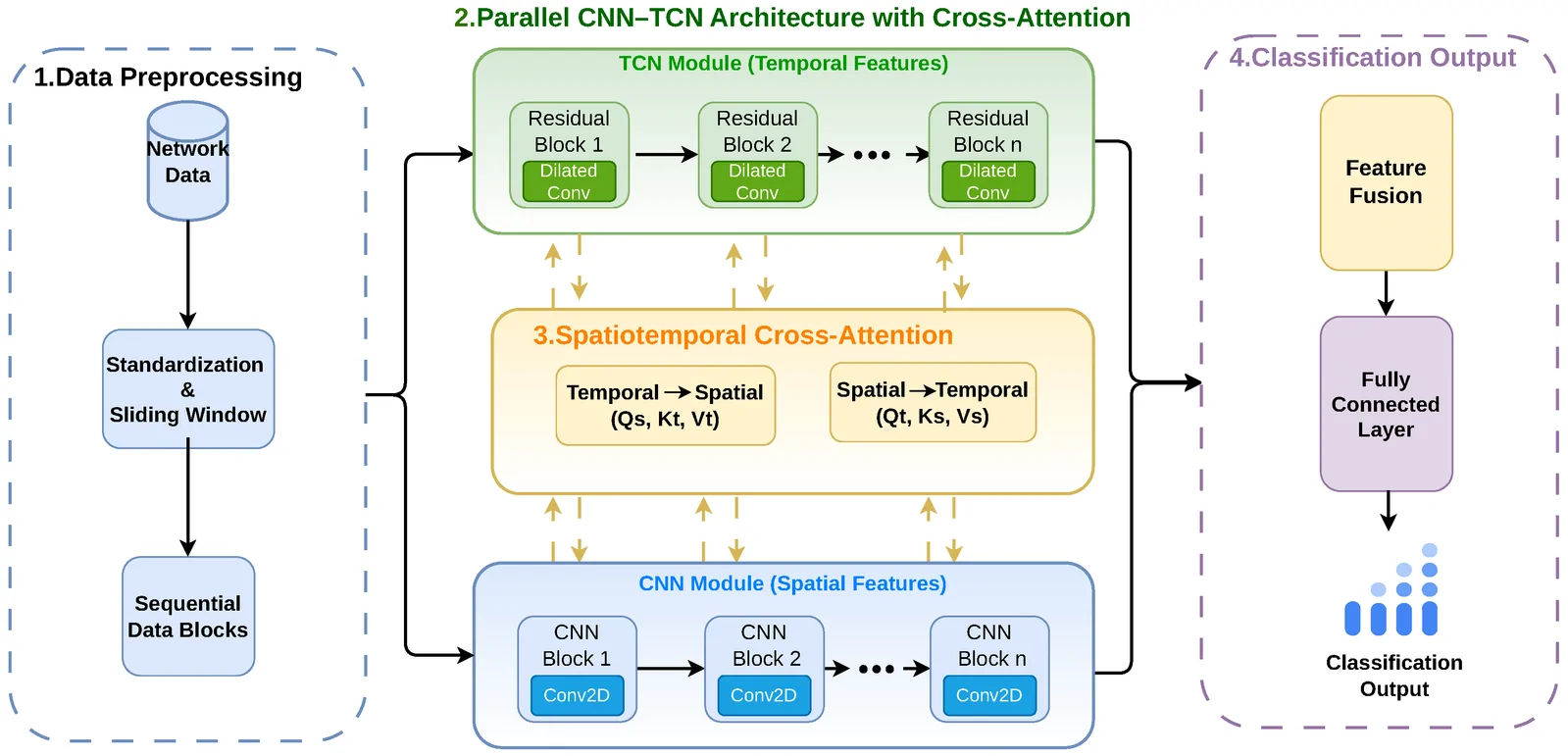
Overall architecture of the proposed TCCA model.

Figure 1 illustrates the overall architecture of TCCA. To preserve contextual relationships among adjacent network events, continuous flow records are organized into fixed-length sequence blocks using an overlapping sliding window.

Given a dataset containing *M* feature-standardized records, a sliding window of length *L* and stride *r* generates a sequence of overlapping blocks. The *j*-th block is defined as

(1) $$X_{j}={\left[x_{jr+1},x_{jr+2},\ldots ,x_{jr+L}\right]}^{⊤}\in R^{L\times I},$$

where $x_{t}\in R^{I}$ denotes the feature vector of the *t*-th traffic record, *I* is the number of selected traffic features, *L* is the window length, and *r* is the sliding stride. The window index is given by $j=0,1,\ldots ,\left\lfloor (M-L)/r\right\rfloor$, where *M* is the number of traffic records in the corresponding data partition.

For batch training with batch size *B*, the input tensor is represented as $X\in R^{B\times L\times I}$. Because a class label is assigned to each traffic record, the corresponding label tensor is denoted by $Y\in {\left\{1,\ldots ,C\right\}}^{B\times L}$, where *C* is the number of traffic classes.

TCCA feeds X into two parallel streams. The CNN branch captures local spatial correlations, while the TCN branch models long-range temporal dependencies. To overcome the representation isolation of conventional parallel architectures, bidirectional cross-attention modules are inserted between intermediate layers. This design enables dynamic feature interaction and refinement before final classification.

### 3.2. Parallel Spatiotemporal Feature Extraction

Unlike serial architectures that suffer from unidirectional feature flow and recurrent latency, TCCA processes spatial and temporal information in parallel. For branch processing, the input tensor is first transposed as $X^{\prime }={\mathrm{Transpose}}_{L,I}\left(X\right)\in R^{B\times I\times L}$.

#### 3.2.1. Spatial Feature Extraction Branch (CNN)

The spatial branch represents each sequence block as a 2D feature map, with traffic features and consecutive records forming its two dimensions. This representation enables convolutional filters to capture local correlations among traffic features and their variations across adjacent time steps. To this end, a channel dimension is first added to the input:

(2) $$X_{s}=\mathrm{Unsqueeze}\left(X^{\prime }\right)\in R^{B\times 1\times I\times L}.$$

The initial inputs of the spatial and temporal branches are defined as ${\tilde{F}}_{s}^{\left(0\right)}=X_{s}$ and ${\tilde{F}}_{t}^{\left(0\right)}=X^{\prime }$, respectively.

**Figure 2:**
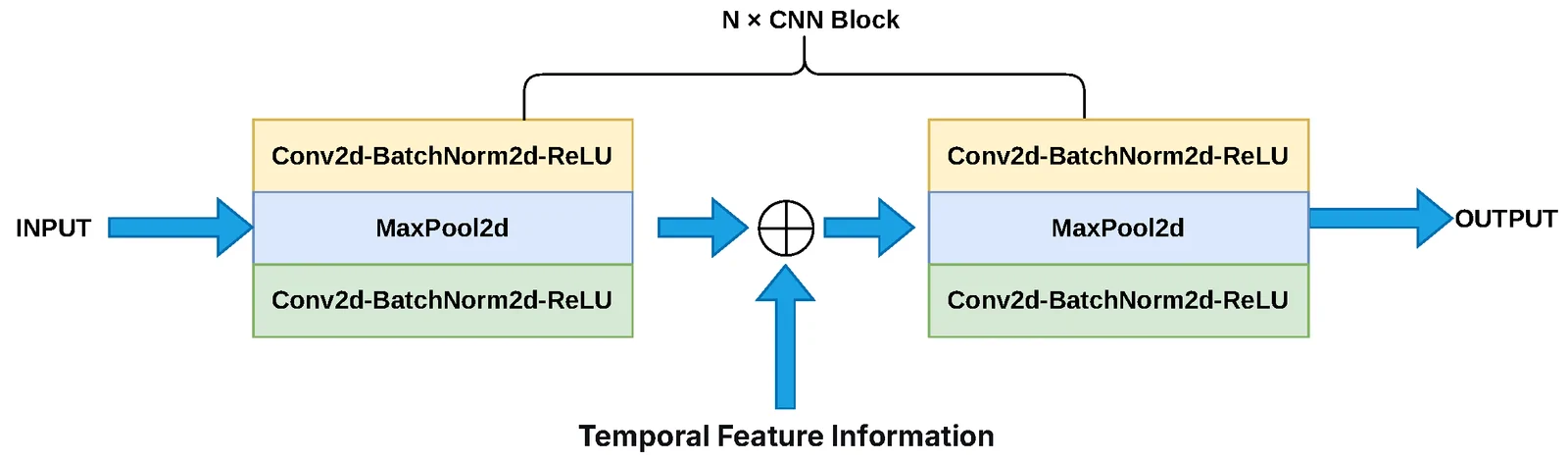
Structure of the CNN spatial feature extraction block.

As shown in Figure 2, the *l*-th CNN block processes the cross-attention-enhanced input ${\tilde{F}}_{s}^{\left(l-1\right)}$ through two 2D convolutional operations:

(3) $$U_{s}^{\left(l\right)}=\mathrm{Pool}\left(\phi \left({\mathrm{BN}}_{1}^{\left(l\right)}\left(W_{s,1}^{\left(l\right)}*{\tilde{F}}_{s}^{\left(l-1\right)}+b_{s,1}^{\left(l\right)}\right)\right)\right),$$

(4) $$F_{s}^{\left(l\right)}=\phi \left({\mathrm{BN}}_{2}^{\left(l\right)}\left(W_{s,2}^{\left(l\right)}*U_{s}^{\left(l\right)}+b_{s,2}^{\left(l\right)}\right)\right),$$

where ∗ denotes 2D convolution, $W_{s,1}^{\left(l\right)}$ and $W_{s,2}^{\left(l\right)}$ are convolution kernel weights, BN(·) denotes batch normalization, ϕ(·) represents the ReLU activation function, and Pool(·) denotes max pooling with a stride of 2.

#### 3.2.2. Temporal Feature Extraction Branch (TCN)

The temporal branch employs stacked TCN residual blocks with dilated causal convolutions to capture long-range temporal dependencies without recurrent computation.

**Figure 3:**
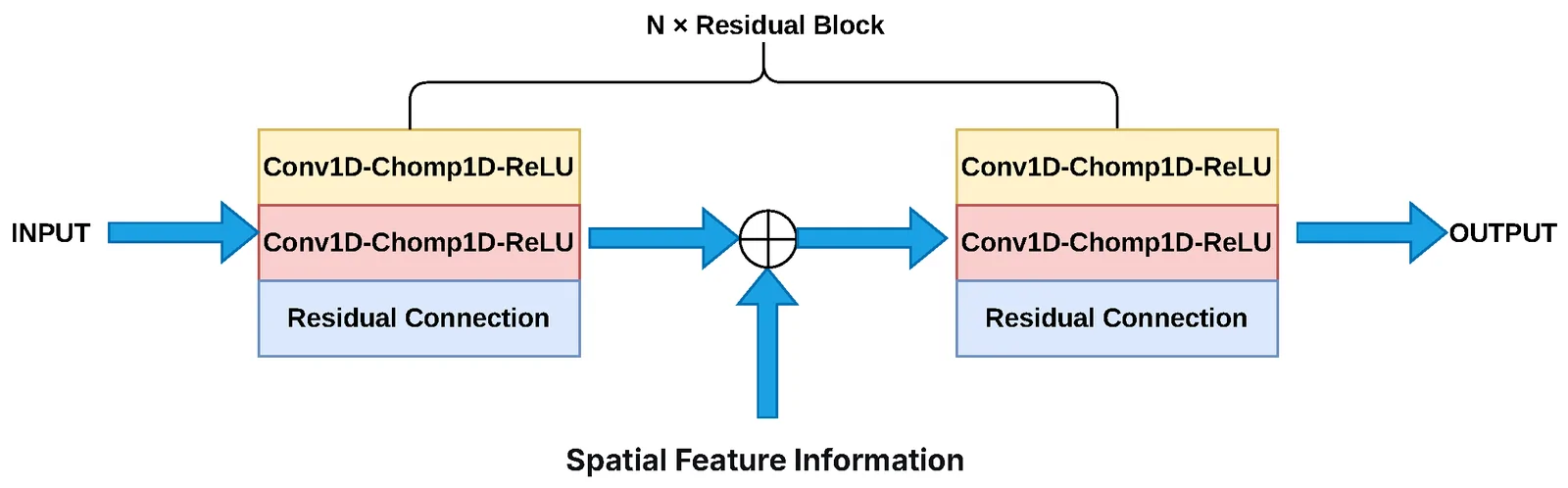
Structure of the TCN temporal feature extraction block.

As shown in Figure 3, the output of the *l*-th TCN block is obtained through residual learning:

(5) $$F_{t}^{\left(l\right)}=\phi \left(F_{t}^{\left(l\right)}\left({\tilde{F}}_{t}^{\left(l-1\right)}\right)+P_{t}^{\left(l\right)}\left({\tilde{F}}_{t}^{\left(l-1\right)}\right)\right),$$

where $F_{t}^{\left(l\right)}\left(\cdot \right)$ denotes the dual dilated-convolution pipeline, and $P_{t}^{\left(l\right)}\left(\cdot \right)$ represents a 1D linear projection used for channel matching.

### 3.3. Inter-Layer Bidirectional Cross-Attention

To overcome feature isolation before late fusion, TCCA inserts bidirectional cross-attention modules between the intermediate CNN and TCN stages. Let $F_{s}^{\left(l\right)}\in R^{B\times C_{l}\times H_{l}\times W_{l}}$ and $F_{t}^{\left(l\right)}\in R^{B\times C_{l}\times L}$ denote the spatial and temporal features at stage *l*, respectively. They are first reshaped into token sequences:

(6) $${\bar{F}}_{s}^{\left(l\right)}=\mathrm{Reshape}\left(F_{s}^{\left(l\right)}\right)\in R^{B\times N_{s}^{\left(l\right)}\times C_{l}},\;\mathrm{where}\;N_{s}^{\left(l\right)}=H_{l}W_{l},$$

(7) $${\bar{F}}_{t}^{\left(l\right)}=\mathrm{Transpose}\left(F_{t}^{\left(l\right)}\right)\in R^{B\times N_{t}\times C_{l}},\;\mathrm{where}\;N_{t}=L.$$

#### 3.3.1. Temporal-to-Spatial Interaction

To enhance spatial representations using temporal context, spatial tokens serve as Queries, while temporal tokens generate Keys and Values:

(8) $$Q_{s}^{\left(l\right)}={\bar{F}}_{s}^{\left(l\right)}W_{s,Q}^{\left(l\right)},\;K_{t}^{\left(l\right)}={\bar{F}}_{t}^{\left(l\right)}W_{t,K}^{\left(l\right)},\;V_{t}^{\left(l\right)}={\bar{F}}_{t}^{\left(l\right)}W_{t,V}^{\left(l\right)}.$$

Similar to the cross-attention mechanism for information exchange between spatial and temporal experts in CAST [33], we formulate our temporal-guided spatial attention as

(9) $$A_{t\to s}^{\left(l\right)}=\mathrm{softmax}\left(\frac{Q_{s}^{\left(l\right)}{\left(K_{t}^{\left(l\right)}\right)}^{⊤}}{\sqrt{d_{l}}}\right)V_{t}^{\left(l\right)},$$

The projection matrices map the channel dimension from $C_{l}$ to $d_{l}$, where $d_{l}=C_{l}$ is used to enable residual addition. The softmax operation is applied along the key-token dimension. Thus, $A_{t\to s}^{\left(l\right)}\in R^{B\times N_{s}^{\left(l\right)}\times C_{l}}$. The spatial representation is updated through a residual connection:

(10) $${\tilde{F}}_{s}^{\left(l\right)}=\mathrm{RestoreShape}\left({\bar{F}}_{s}^{\left(l\right)}+A_{t\to s}^{\left(l\right)}\right)\in R^{B\times C_{l}\times H_{l}\times W_{l}}.$$

#### 3.3.2. Spatial-to-Temporal Interaction

Conversely, temporal features query the spatial representations to identify important local feature correlations:

(11) $$Q_{t}^{\left(l\right)}={\bar{F}}_{t}^{\left(l\right)}W_{t,Q}^{\left(l\right)},\;K_{s}^{\left(l\right)}={\bar{F}}_{s}^{\left(l\right)}W_{s,K}^{\left(l\right)},\;V_{s}^{\left(l\right)}={\bar{F}}_{s}^{\left(l\right)}W_{s,V}^{\left(l\right)}.$$

The spatial-guided temporal attention is defined as

(12) $$A_{s\to t}^{\left(l\right)}=\mathrm{softmax}\left(\frac{Q_{t}^{\left(l\right)}{\left(K_{s}^{\left(l\right)}\right)}^{⊤}}{\sqrt{d_{l}}}\right)V_{s}^{\left(l\right)},$$

and the enhanced temporal features are obtained as

(13) $${\tilde{F}}_{t}^{\left(l\right)}=\mathrm{TransposeBack}\left({\bar{F}}_{t}^{\left(l\right)}+A_{s\to t}^{\left(l\right)}\right)\in R^{B\times C_{l}\times L}.$$

Here, the softmax operation is again applied along the key-token dimension, giving $A_{s\to t}^{\left(l\right)}\in R^{B\times N_{t}\times C_{l}}$. This layer-wise mutual guidance enables the spatial and temporal streams to continuously exchange information and refine each other throughout feature learning.

### 3.4. Feature Fusion and Classification

After the final interaction stage, let $N_{s}=N_{s}^{\left(N\right)}=H_{N}W_{N}$ denote the number of spatial tokens. The enhanced spatial feature tensor is then reshaped and transposed to $S^{\left(N\right)}\in R^{B\times C_{N}\times N_{s}}$. Because the temporal convolutions preserve the sequence length, $N_{t}=L$ throughout the network. As the spatial and temporal branches contain different numbers of tokens, a learned linear projection $P_{s}:R^{N_{s}}\to R^{N_{t}}$ aligns the spatial token dimension with the temporal sequence length:

(14) $$F_{s}^{\prime }=P_{s}\left(S^{\left(N\right)}\right)\in R^{B\times C_{N}\times N_{t}}.$$

The aligned spatial features are then fused with the enhanced temporal features by element-wise addition:

(15) $$F=F_{s}^{\prime }+{\tilde{F}}_{t}^{\left(N\right)}\in R^{B\times C_{N}\times N_{t}}.$$

Finally, a shared time-wise linear classifier maps the fused features to class logits $Z\in R^{B\times N_{t}\times C}$, and the class with the highest logit is selected for each traffic record during inference.

The complete forward propagation procedure is presented in Algorithm 1.
**Algorithm 1** Forward Propagation of TCCA**Require:** 
Sequence block tensor $X\in R^{B\times L\times I}$**Ensure:** 
Class predictions $\hat{Y}\in {\left\{1,\ldots ,C\right\}}^{B\times L}$1:$X^{\prime }\leftarrow {\mathrm{Transpose}}_{L,I}\left(X\right)$2:${\tilde{F}}_{s}^{\left(0\right)}\leftarrow \mathrm{Unsqueeze}\left(X^{\prime }\right)$, $\;{\tilde{F}}_{t}^{\left(0\right)}\leftarrow X^{\prime }$3:**for** l=1 **to** *N* **do**4:   $F_{s}^{\left(l\right)}\leftarrow {\mathrm{CNNBlock}}_{l}\left({\tilde{F}}_{s}^{\left(l-1\right)}\right)$5:   $F_{t}^{\left(l\right)}\leftarrow {\mathrm{TCNBlock}}_{l}\left({\tilde{F}}_{t}^{\left(l-1\right)}\right)$6:   Flatten/transpose $F_{s}^{\left(l\right)}$ and $F_{t}^{\left(l\right)}$ into token sequences ${\bar{F}}_{s}^{\left(l\right)}$ and ${\bar{F}}_{t}^{\left(l\right)}$7:   Compute $Q_{s}^{\left(l\right)},K_{s}^{\left(l\right)},V_{s}^{\left(l\right)}$ and $Q_{t}^{\left(l\right)},K_{t}^{\left(l\right)},V_{t}^{\left(l\right)}$8:   $A_{t\to s}^{\left(l\right)}\leftarrow \mathrm{CrossAttention}\left(Q_{s}^{\left(l\right)},K_{t}^{\left(l\right)},V_{t}^{\left(l\right)}\right)$9:   $A_{s\to t}^{\left(l\right)}\leftarrow \mathrm{CrossAttention}\left(Q_{t}^{\left(l\right)},K_{s}^{\left(l\right)},V_{s}^{\left(l\right)}\right)$10:   ${\tilde{F}}_{s}^{\left(l\right)}\leftarrow \mathrm{RestoreShape}\left({\bar{F}}_{s}^{\left(l\right)}+A_{t\to s}^{\left(l\right)}\right)$11:   ${\tilde{F}}_{t}^{\left(l\right)}\leftarrow \mathrm{TransposeBack}\left({\bar{F}}_{t}^{\left(l\right)}+A_{s\to t}^{\left(l\right)}\right)$12:**end for**13:Reshape/transpose ${\tilde{F}}_{s}^{\left(N\right)}$ into $S^{\left(N\right)}\in R^{B\times C_{N}\times N_{s}}$14:$F_{s}^{\prime }\leftarrow P_{s}\left(S^{\left(N\right)}\right)$15:$F\leftarrow F_{s}^{\prime }+{\tilde{F}}_{t}^{\left(N\right)}$16:Z←FC(Transpose(F))17:$\hat{Y}\leftarrow {arg max}_{c}Z_{:,:,c}$18:**return** $\hat{Y}$

## 4. Experiments and Analysis

This section evaluates TCCA in terms of overall classification performance, detection of minority-class and difficult-to-detect attacks, effectiveness of inter-layer cross-attention, computational efficiency, and sensitivity to temporal structure. Experiments were conducted on the CICIDS-2017 and CIC IIoT 2025 datasets, which cover diverse attack categories and provide network traffic sequences for temporal analysis.

### 4.1. Experimental Setup

#### 4.1.1. Datasets and Data Partitioning

We evaluated TCCA on two network security benchmarks: CICIDS-2017 [34], which contains benign traffic and common network attacks grouped into six primary classes after excluding the extremely rare Infiltration and Heartbleed categories, and CIC IIoT 2025 [35], which represents operational IoT/IIoT traffic across eight distinct classes without class merging or removal. Table 2 and Table 3 summarize the class distributions of the processed datasets.

Both datasets exhibit substantial class imbalance. In CICIDS-2017, Web Attack and Bot account for only 0.0771% and 0.0692% of the samples, respectively. These distributions provide a challenging setting for evaluating TCCA across diverse attack categories, particularly minority-class attacks.

To prevent data leakage, source and destination IP addresses, ports, and composite flow identifiers were excluded from the model inputs. Absolute timestamps were used only for chronological ordering and temporal partitioning. Each dataset was divided into training, validation, and test sets before data-dependent preprocessing, oversampling, and sliding window construction. For CICIDS-2017, records within each day and class were chronologically ordered and split at a 6:2:2 ratio. For CIC IIoT 2025, hierarchical stratification was applied to preserve the class distribution, followed by a temporal cutoff that reserves the final time window exclusively for testing.

Missing and infinite values were replaced with feature medians computed from the training set, followed by Z-score standardization fitted on the training data. The same transformations and 68 features selected by ExtraTrees (listed in Supplementary Tables S1 and S2) were applied to the validation and test sets in a fixed order. SMOTE was applied only to the training splits in closed-set experiments and was excluded from the strict temporal evaluation on CIC IIoT 2025. Validation and test sequences were constructed using non-overlapping windows of length L=32, ensuring that each evaluated record was counted only once.

#### 4.1.2. Implementation Details and Metrics

All experiments were conducted under Windows 11 using PyTorch 2.8, with an Intel Core i5-12400F CPU, 32 GB RAM, and an NVIDIA GeForce RTX 4060 GPU with 8 GB VRAM. For feature selection, ExtraTrees used 300 trees, balanced class weights, all CPU cores, and up to 100,000 training flows per class (seed 42). The top 68 features were retained in their original column order. Training-only SMOTE (k=5, seed 42) increased Bot/Web Attack counts from 1172/1308 to 1500/1800 on CICIDS-2017 and WEB/BRUTEFORCE counts from 5424/3610 to 6000/4000 on CIC IIoT 2025. Other classes were unchanged, and SMOTE was omitted from the strict temporal evaluation. Both datasets used at most 100 training epochs, with early stopping on validation macro-F1 (minimum 20 epochs, patience 10, $\min\_\mathrm{delta}=10^{-4}$); the best validation checkpoint was retained.

As shown in Table 4, the TCN branch uses 32 and 64 channels with dilation factors of 1 and 2, respectively, while the CNN branch employs channel mappings of 1→64→32 and 32→64→64, respectively. The model is trained using AdamW with a batch size of 32, a dropout rate of 0.5, and an initial learning rate of 0.001, which decays by a factor of 0.7 every 10 epochs. The hyperparameters of Equalization Loss v2 (EQL v2) [36] were optimized through a 48-combination grid search on the validation set, with γ∈{10,12,14,16}, μ∈{0.60,0.65,0.70,0.75}, and α∈{6,8,10}. The optimal configuration was γ=12, μ=0.70, and α=10.

Given the substantial class imbalance, model performance was evaluated using overall accuracy together with macro-averaged Precision, Recall, and F1-score. Class-specific metrics are additionally reported for minority-class and difficult-to-detect attacks, including Web Attack and Bot. To assess practical deployment capability, per-record inference latency and flow throughput were also measured.

#### 4.1.3. Baseline Models and Comparison Protocol

To evaluate TCCA against different feature-learning architectures, four representative baseline models were reproduced using the same data partitions, selected features, preprocessing procedures, and labeling protocol. CNN [37] serves as a single-branch feature extraction baseline. CNN-LSTM [38] combines convolutional layers with bidirectional recurrent layers to process individual flow representations. CNN-GRU, adapted from Appathurai et al. [39], combines convolutional feature extraction with GRU-based temporal modeling. LCT-IDS [29] represents a lightweight cascaded architecture that integrates CNN-based local feature extraction with a compact Transformer.

CNN, CNN-LSTM, and LCT-IDS retain their original single-flow input configurations, whereas CNN-GRU and TCCA both process temporally ordered windows of 32 flow records. This setup enables a direct comparison between CNN-GRU and TCCA under the same temporal context, while the single-flow baselines provide additional architectural references.

To ensure a fair comparison, the hyperparameters of all baseline models were independently optimized using Optuna on the validation set, with macro-F1 as the optimization objective. The Tree-structured Parzen Estimator (TPE) sampler was used to conduct 20 trials for each model. The test set was excluded from hyperparameter selection and used only for final evaluation. All models shared identical data partitions, feature selection, and preprocessing procedures.

The common Optuna search space included the learning rate ($3\times 10^{-4}$–$5\times 10^{-3}$, log scale), weight decay ($10^{-6}$–$10^{-3}$, log scale), and dropout rate (0–0.7), where applicable. Architecture-specific search spaces and the selected configurations are summarized in Table 5. For each baseline, the configuration yielding the highest validation macro-F1 was selected for the final evaluation. Hyperparameter selection was performed exclusively on the validation set, and the held-out test set was evaluated only after the optimal configuration had been fixed. This protocol reduces the possibility that the reported baseline performance would be affected by unfavorable default configurations or test-set-guided parameter selection.

### 4.2. Overall Performance Comparison

#### 4.2.1. Convergence Analysis

**Figure 4:**
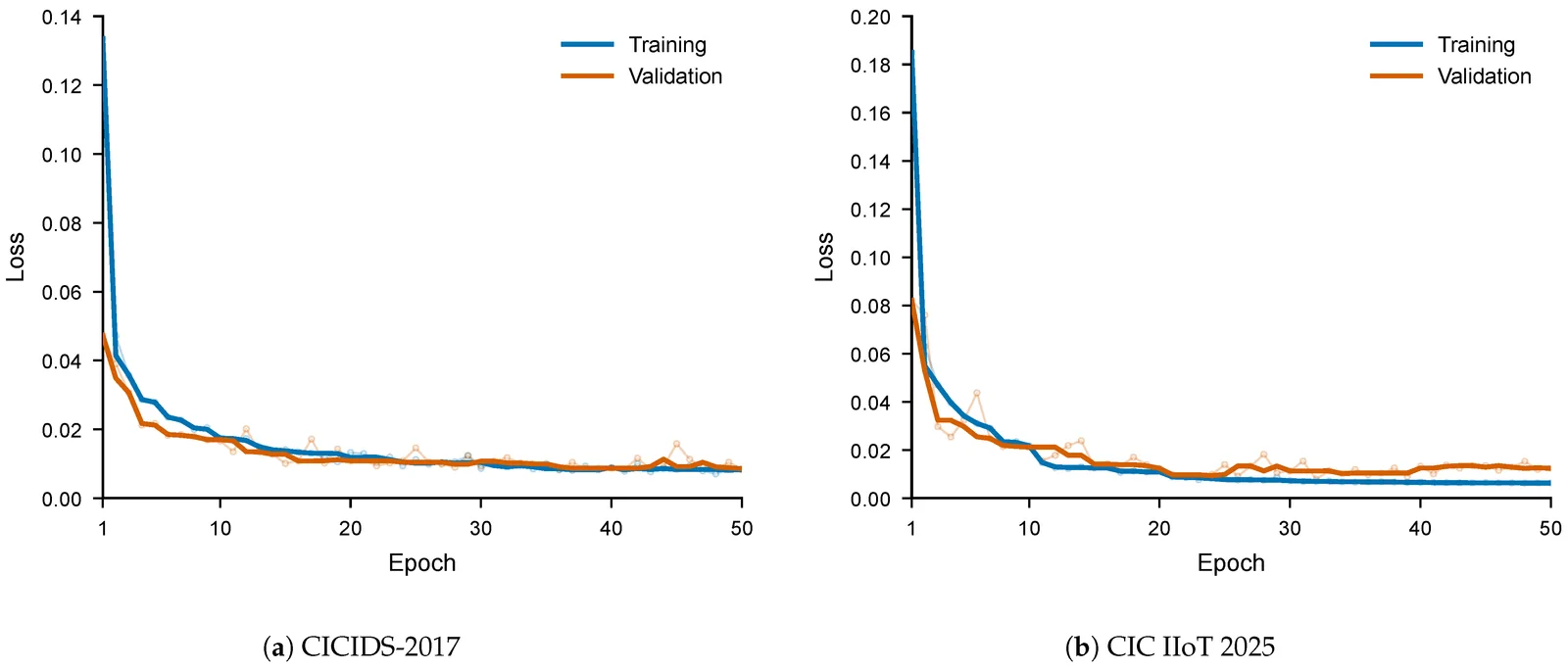
Training and validation loss convergence on (**a**) CICIDS-2017 and (**b**) CIC IIoT 2025. Faint points and lines represent the raw per-epoch losses, while the solid curves denote the five-epoch moving median (with the first point anchored to the first epoch). The horizontal axis spans epochs 1 to 50, and the vertical axis uses a linear loss scale with a major-tick interval of 0.02. Smoothing is applied only for visual trend estimation.

Figure 4 presents the training and validation loss trajectories of TCCA over 50 epochs. On both datasets, the losses decrease rapidly during approximately the first 10 epochs and then decline more gradually. The curves become relatively stable after approximately 20 epochs, indicating effective convergence. On CICIDS-2017, the training and validation losses remain closely aligned despite several small fluctuations in the validation loss. On CIC IIoT 2025, the validation loss increases slightly during the later epochs. while the training loss remains low, indicating mild overfitting. Therefore, an early stopping strategy based on validation performance is used to select the final model.

#### 4.2.2. Overall Multiclass Performance

To evaluate the overall detection performance of TCCA, we compared it with four baseline models using the same data partitions and preprocessing procedures. Table 6 and Table 7 report the mean and sample standard deviation of five independent runs.

On CICIDS-2017, TCCA achieves the highest mean accuracy (99.89%) and macro-F1 (94.49%), as shown in Table 6. Its accuracy exceeds that of LCT-IDS by 0.56 percentage points, while its macro-F1 exceeds that of CNN by 6.94 percentage points. TCCA also achieves macro precision and recall of 94.46% and 94.97%, respectively. Two-sided paired *t*-tests indicate significant improvements in macro-F1 over all baselines (p≤0.0081).

On CIC IIoT 2025, TCCA achieves the highest mean accuracy (99.81%), macro precision (99.70%), macro recall (99.54%), and macro-F1 (99.62%), as shown in Table 7. Its macro-F1 exceeds those of the baselines by 5.21–6.54 percentage points, with statistically significant differences (p<0.001). Overall, the results on both datasets demonstrate the effectiveness of TCCA for multiclass intrusion detection. The improvements are consistent with the benefits of inter-layer bidirectional cross-attention, which enables information exchange between the parallel CNN and TCN branches during feature extraction.

### 4.3. Detection Performance for Individual Attack Classes

#### 4.3.1. Class-Specific Performance of TCCA

Aggregate metrics can obscure performance variations across individual attack categories. Table 8 and Table 9 therefore report the class-specific Precision, Recall, and F1-score of TCCA on the two benchmark datasets.

As shown in Table 8, TCCA achieves consistently strong performance on the major traffic categories in CICIDS-2017. BENIGN, DoS, Patator, and Port Scan all achieve F1-scores above 99%. For Bot traffic, TCCA attains a recall of 96.68% and an F1-score of 92.53%, indicating strong sensitivity to this minority attack category. Web Attack remains the most challenging class. It accounts for only 0.0771% of the dataset and exhibits relatively subtle application-layer characteristics. Nevertheless, TCCA achieves a precision of 86.28%, a recall of 75.00%, and an F1-score of 80.25%. These results indicate that TCCA maintains meaningful detection capability even under severe class imbalance. The combination of SMOTE and EQL v2 helps reduce the dominance of majority classes during training and improves the representation of underrepresented attack categories.

On CIC IIoT 2025, TCCA achieves consistently high performance across all eight traffic categories, as shown in Table 9. Seven of the eight classes obtain F1-scores above 99%. Even the minority BRUTEFORCE class, which accounts for only 0.88% of the dataset, achieves a precision of 99.16%, a recall of 98.17%, and an F1-score of 98.66%. The WEB category also achieves a precision of 99.78%, a recall of 99.72%, and an F1-score of 99.75%. These results indicate that TCCA maintains stable discrimination across both majority and minority traffic categories on the IIoT benchmark.

#### 4.3.2. Comparison on Minority and Difficult-to-Detect Attacks

To assess detection performance under class imbalance, Table 10 and Table 11 compare the class-specific F1-scores of TCCA and the baseline models, with particular attention to minority-class and difficult-to-detect attacks.

The class-specific results in Table 10 further demonstrate the performance differences among the evaluated models on CICIDS-2017. For Web Attack, the baseline F1-scores range from 22.18% for LCT-IDS to 70.35% for CNN-LSTM. TCCA achieves an F1-score of 80.25%, outperforming the strongest baseline by 9.90 percentage points. This improvement is notable because Web Attack accounts for only 0.0771% of the processed dataset. TCCA achieves the highest F1-score in four of the six classes: BENIGN, DoS, Port Scan, and Web Attack. For Bot and Patator, its F1-score is 0.53 and 0.19 percentage points lower than those of LCT-IDS and CNN-GRU, respectively. Overall, TCCA provides a comparatively balanced class-specific performance profile, with its largest advantage observed for the severely underrepresented Web Attack class.

As shown in Table 11, TCCA achieves the highest F1-score across all eight traffic categories. The largest gains are observed for BRUTEFORCE and RECON. For BRUTEFORCE, TCCA achieves an F1-score of 98.66%, exceeding the strongest baseline, CNN-GRU, by 9.33 percentage points. For RECON, TCCA reaches 99.76%, outperforming CNN-GRU by 8.17 percentage points. Compared with the strongest baseline result for each class, TCCA also improves the F1-score for WEB, DDoS, MITM, and MALWARE by 5.25, 4.27, 4.07, and 3.52 percentage points, respectively. TCCA achieves improvements of 3.10 and 2.29 percentage points for BENIGN and DoS, respectively. These results indicate that TCCA maintains consistently strong class-specific performance across the evaluated IIoT traffic categories, including the underrepresented BRUTEFORCE and WEB classes.

Overall, the class-level results on both datasets indicate that TCCA provides more consistent detection across traffic categories rather than improving performance only on dominant classes. The strongest improvement is observed for the highly imbalanced Web Attack class in CICIDS-2017, where TCCA substantially reduces the performance gap between minority and majority classes. This behavior is consistent with the proposed architecture, in which the parallel CNN and TCN branches capture complementary spatial and temporal patterns, while inter-layer bidirectional cross-attention enables these representations to exchange information throughout feature extraction.

#### 4.3.3. Confusion Matrix Analysis

**Figure 5:**
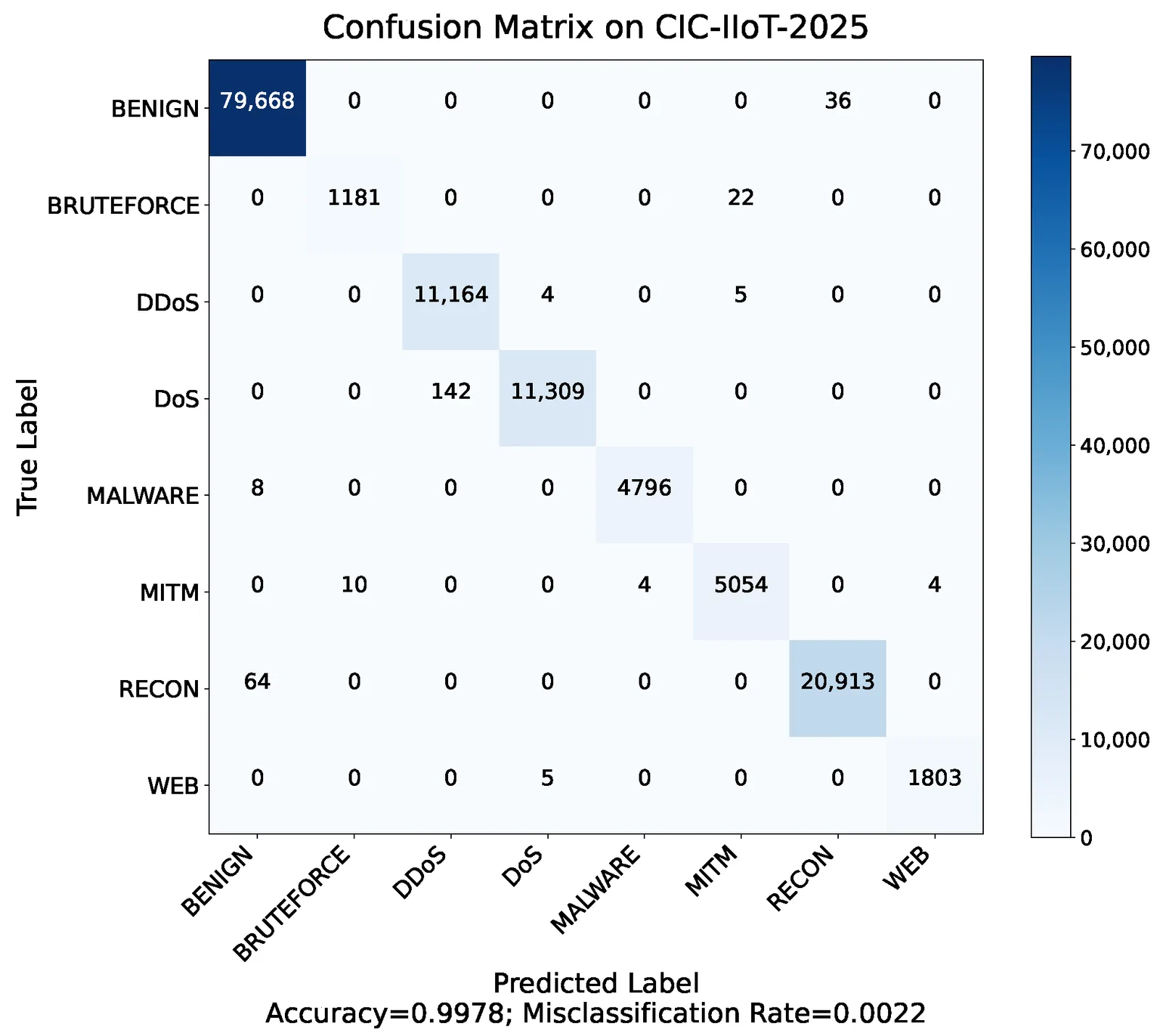
Confusion matrix of the proposed TCCA model on CIC IIoT 2025.

**Figure 6:**
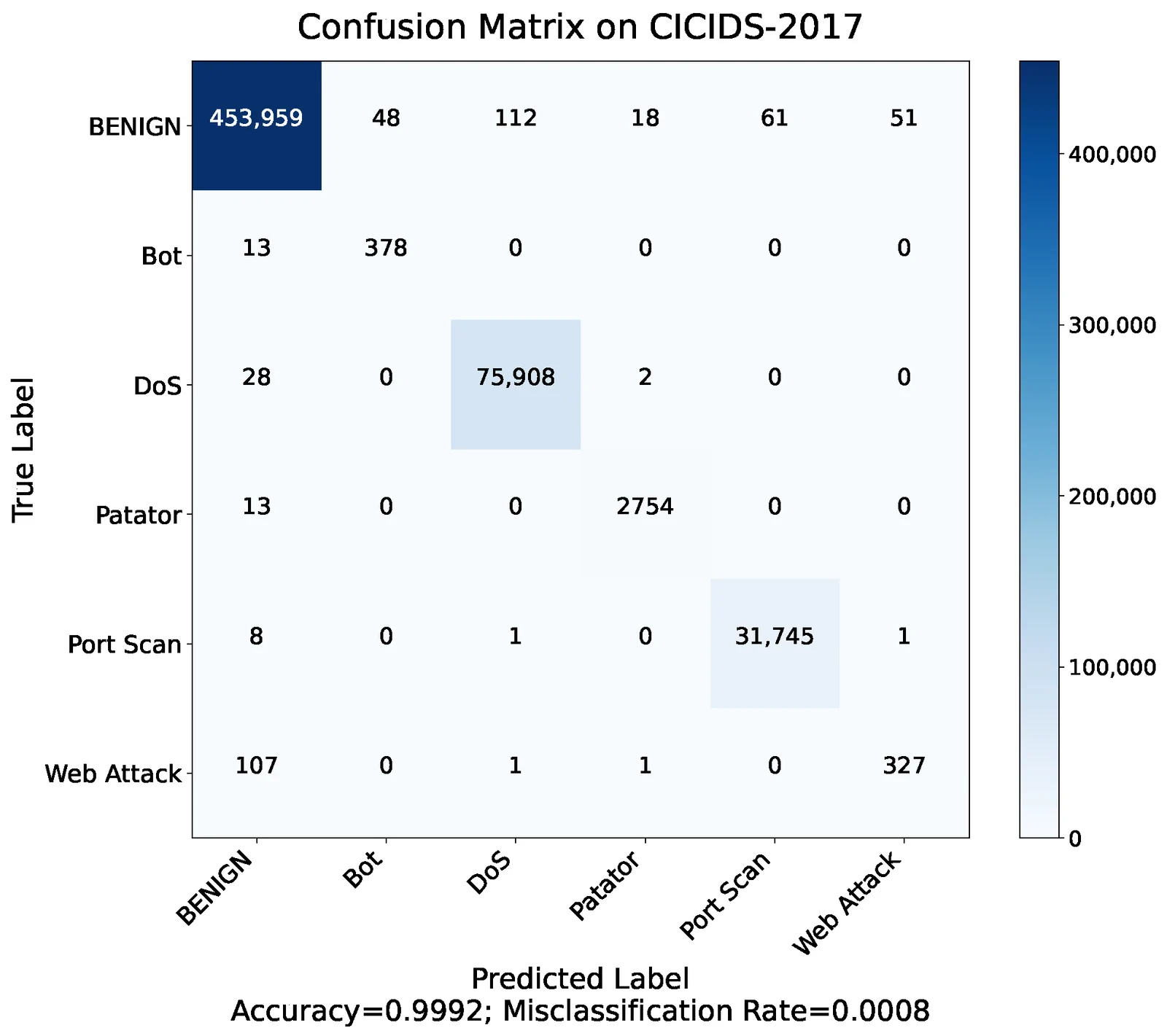
Confusion matrix of the proposed TCCA model on CICIDS-2017.

To complement the macro-averaged metrics and provide a detailed view of class-level misclassification patterns, Figure 5 and Figure 6 present the confusion matrices of TCCA on the test sets of CIC IIoT 2025 and CICIDS-2017, respectively.

On CICIDS-2017 (Figure 6), the confusion matrix shows strong diagonal dominance across most traffic categories, consistent with the high class-specific precision and recall reported in Table 8. The main source of error is Web Attack traffic. Of the 436 Web Attack instances, 327 are correctly classified, 107 are misclassified as BENIGN, and the remaining two are assigned to other attack classes. This results in a recall of 75.00% and an F1-score of 80.25%. This error concentration is likely related to two factors: (1) the extreme scarcity of Web Attack samples, which account for only 0.0771% of the dataset, and (2) the flow-level similarity between application-layer attacks, such as SQL injection and XSS, and legitimate HTTP traffic. These characteristics make the Web Attack class more difficult to distinguish from benign traffic than the other attack categories.

In contrast, the confusion matrix for CIC IIoT 2025 (Figure 5) exhibits near-diagonal predictions with limited off-diagonal errors. This observation is consistent with the high class-specific F1-scores reported in Table 9, where seven of the eight classes achieve F1-scores above 99%, while BRUTEFORCE achieves an F1-score of 98.66%. The substantially lower misclassification rate suggests that TCCA can effectively distinguish different traffic categories in this dataset. The inter-layer spatiotemporal cross-attention mechanism enables spatial and temporal representations to exchange information during feature extraction, which may contribute to clearer class separation. However, the specific contribution of protocol characteristics to this performance cannot be established from the confusion matrix alone and would require additional controlled analysis.

### 4.4. Ablation Study

To examine the contributions of cross-attention, fusion depth, and SMOTE, we evaluated seven model variants. CNN–TCN removes cross-attention and directly adds the outputs of the two branches. TCCA-T→S and TCCA-S→T retain only one attention direction, where T→S uses temporal features to update spatial features. First-layer fusion only and Last-layer fusion only apply bidirectional cross-attention at only the first or last stage, respectively. TCCA w/o SMOTE removes training-set oversampling, while Full TCCA uses bidirectional cross-attention at both stages and SMOTE. All variants use the same data partitions and preprocessing procedures, except for the SMOTE ablation. Results are reported as mean ± sample standard deviation over five seeds: 42, 123, 2024, 3407, and 8888.

As shown in Table 12, Full TCCA achieves the highest macro-F1 on both datasets. On CICIDS-2017, its macro-F1 reaches 94.49±0.94%, exceeding CNN–TCN by 5.15 percentage points. Both single-direction variants improve upon CNN–TCN, but neither matches Full TCCA. Last-layer fusion also performs better than first-layer fusion (92.47% versus 91.63%). Removing SMOTE reduces macro-F1 to 93.74%. On CIC IIoT 2025, Full TCCA achieves a macro-F1 of 99.62±0.13%, compared with 99.32±0.06% for CNN–TCN. The smaller gain is consistent with the already high performance of CNN–TCN on this dataset. The single-direction and single-layer variants also achieve lower macro-F1 than Full TCCA. Removing SMOTE reduces macro-F1 slightly to 99.56%. Overall, these results support the use of bidirectional cross-attention at multiple stages. SMOTE provides a larger improvement on CICIDS-2017 than on CIC IIoT 2025.

**Figure 7:**
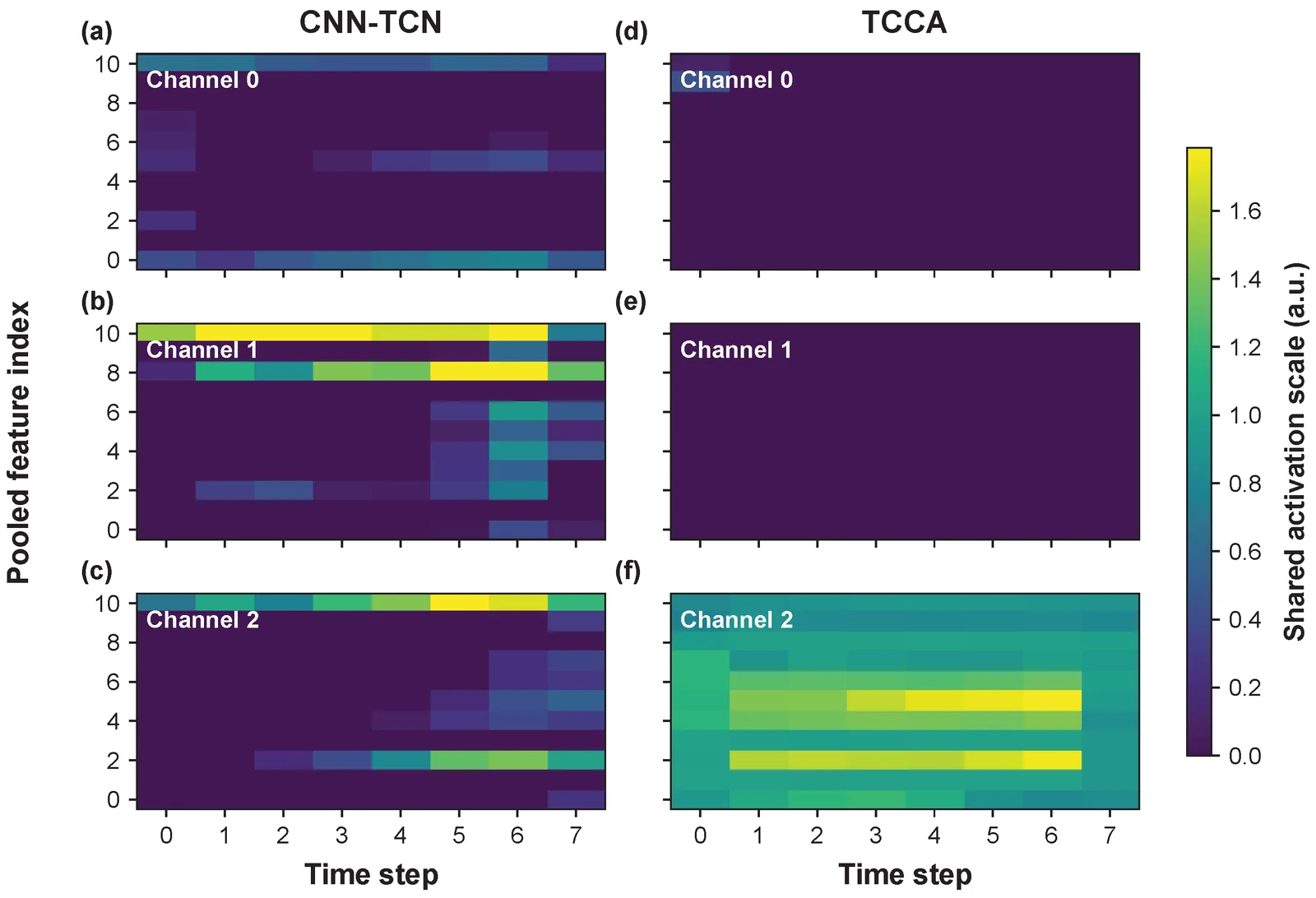
Cross-attention is associated with differentiated CNN channel responses. Heatmaps show the first three channels of the second CNN layer for CNN–TCN (**a**–**c**) and TCCA (**d**–**f**), using the first sample from evaluation batch 112. All panels show raw activations on a shared colour scale, with values above the global 99th percentile clipped for visualization only.

To further examine the effect of cross-attention at the representation level, Figure 7 compares the feature activations of the second CNN layer in CNN–TCN and TCCA. The channels in CNN–TCN show relatively similar activation patterns across spatial locations (Figure 7a–c), whereas TCCA produces more differentiated activation patterns across channels (Figure 7d–f). This observation is consistent with the quantitative results in Table 12 and suggests that cross-attention promotes more diverse feature representations and reduces channel redundancy. These heatmaps provide qualitative evidence of structural differences in the learned representations rather than explicit semantic attribution to specific attack stages.

Table 13 provides a quantitative comparison of the learned representations. Reported means were calculated across bootstrap resamples, and evaluation was performed on the held-out test set. TCCA reduces mean absolute channel correlation from 0.2411 to 0.2349 and increases normalized effective rank from 0.2397 to 0.2582. These results suggest a modest reduction in channel redundancy. TCCA also achieves a higher Fisher ratio (3.6169 versus 1.7506) and Silhouette score (0.4794 versus 0.4040), along with a lower Davies–Bouldin index (0.7614 versus 0.9296). These changes indicate better class separability. The cross-model CKA score of the CNN representations is 0.8587±0.0041 (95% CI: [0.8513,0.8669]), indicating substantial similarity between the two models. Although TCCA achieves a higher linear-probe macro-F1, the difference is not statistically significant (p=0.244). Together, these findings suggest that cross-attention improves several representation properties.

### 4.5. Computational Efficiency Analysis

Inference latency, throughput, and resource usage are important for IIoT intrusion detection. To evaluate computational efficiency, all models are tested on the same hardware using batches of 1024 traffic records. CNN, CNN-LSTM, and LCT-IDS process 1024×68 single-flow inputs, while CNN-GRU and TCCA process 32 windows of 32 consecutive records. Thus, each model processes the same number of raw records per batch. After a warm-up phase, inference latency is measured over 50 runs with gradient computation disabled. Computational End-to-End (E2E) latency also includes preprocessing, window construction, device transfer, and output post-processing. Per-flow latency is obtained by dividing batch latency by 1024, while throughput is reported in flows/s.

As shown in Table 14, TCCA achieves an inference latency of 0.0034 ms/flow and a computational E2E latency of 0.0037 ms/flow, with an inference throughput of 297,033.55 flows/s. Its inference latency is 87.36%, 84.55%, and 63.44% lower than that of CNN-GRU, CNN-LSTM, and LCT-IDS, respectively. Under the equivalent workload of 1024 raw flows, TCCA requires 2.03 GFLOPs and 47.32 MiB of peak GPU memory. Although CNN remains faster because of its simpler architecture, TCCA provides a favorable balance between detection performance and computational efficiency.

### 4.6. Parameter Sensitivity and Robustness Analysis

To further evaluate the operational robustness and stability of TCCA under diverse deployment conditions, this section examines five aspects: (1) sensitivity to the sequence length (*L*), (2) computational complexity, (3) training stability across random seeds, (4) unseen-attack detection under temporal distribution shifts, and (5) robustness to input noise and random masking.

#### 4.6.1. Effect of Sequence Length

To determine an appropriate temporal window size, we evaluated L∈{8,16,32,64} on CIC IIoT 2025 while keeping the remaining hyperparameters unchanged. All experiments used random seed 123. Table 15 reports the accuracy, Macro-F1, and per-sequence inference latency.

Increasing *L* from 8 to 32 improved the accuracy from 99.23% to 99.78% and the Macro-F1 from 97.97% to 99.53%. This result indicates that shorter windows may provide insufficient temporal context. Increasing *L* to 64 slightly reduced the accuracy and Macro-F1 to 99.58% and 99.42%, respectively, while increasing the sequence latency from 0.14 ms to 0.21 ms. Therefore, L=32 provided the best balance between detection performance and inference latency.

#### 4.6.2. Computational Complexity Analysis

TCCA uses parallel CNN and TCN branches to extract spatial and temporal features without recurrent dependencies. For a convolutional layer, the computational complexity depends on the feature-map size, kernel size, and channel dimensions. The CNN branch has a complexity of $O(N_{s}k_{s}^{2}C_{s}^{2})$ for two-dimensional convolution, while the TCN branch has a complexity of $O(N_{t}k_{t}C_{t}^{2})$ for one-dimensional convolution, assuming comparable input and output channel dimensions. For each cross-attention stage, let $N_{s}$ and $N_{t}$ denote the numbers of spatial and temporal tokens, respectively, and let *d* be the embedding dimension. The linear projections require $O(\left(N_{s}+N_{t}\right)d^{2})$ operations, while bidirectional cross-attention requires $O(2N_{s}N_{t}d)$. Feature fusion and classification introduce additional computation that depends on the corresponding feature dimensions. Unlike recurrent architectures, the convolutional and attention operations in TCCA can be parallelized across temporal positions. However, bidirectional cross-attention introduces a quadratic interaction term with respect to token counts when $N_{s}$ and $N_{t}$ grow together. Therefore, the computational cost depends on the sequence length, feature dimensions, and number of interaction stages. This analysis explains the main sources of computational overhead in TCCA, while the measured efficiency is reported in Section 4.5.

#### 4.6.3. Stability Across Random Seeds

After identifying L=32 as the optimal sequence length, we further evaluated the training stability and reproducibility of TCCA under stochastic variations in parameter initialization and mini-batch sampling. Experiments were repeated using five independent random seeds (42, 123, 2024, 3407, and 8888) on both CICIDS-2017 and CIC IIoT 2025, with identical preprocessing and hyperparameter settings.

Table 16 reports the mean and standard deviation across the five independent runs. TCCA shows stable performance on both benchmarks, with an accuracy of 99.89%±0.05% on CICIDS-2017 and 99.81%±0.13% on CIC IIoT 2025. The small standard deviations indicate that the model is relatively robust to variations in random initialization and mini-batch sampling. CICIDS-2017 shows a larger variation in macro-F1, with a standard deviation of 0.94%. This variation is mainly associated with the extreme class imbalance of minority attack categories, for which the learned decision boundaries may be more sensitive to stochastic training conditions.

#### 4.6.4. Attack-Temporal Generalization Evaluation

While closed-set evaluation focuses on known attack categories, real-world intrusion detection systems must also handle out-of-distribution (OOD) traffic caused by temporal distribution shifts and previously unseen attacks. To evaluate the unseen-attack generalization capability of TCCA, we constructed an attack-temporal split benchmark on CIC IIoT 2025.

All attack sub-categories were merged into a single ATTACK class, resulting in a binary BENIGN-vs-ATTACK classification task. Traffic collected from 15–21 January and 23–27 January 2025 was used for training and validation, respectively, while traffic from 28 January to 12 June 2025 was reserved for testing. The training set contained only DoS and RECON attacks. DDoS and MITM were absent from the training set but appeared in both the validation and test sets, whereas BRUTEFORCE, MALWARE, and WEB appeared exclusively in the test set and remained unseen during both training and validation. Scaling parameters were fitted using the training set only, and sequence windows (L=32) were constructed within individual devices and days to prevent cross-boundary data leakage.

As shown in Table 17, TCCA achieves the highest accuracy (92.00%) and macro-F1 (91.66%) under the temporal split. It also achieves the highest attack precision (97.10%) and attack F1-score (89.98%). Its false-positive rate is only 1.88%, compared with 21.20% for CNN-GRU and 17.40% for LCT-IDS. However, TCCA has a lower attack recall (83.83%) than both baselines, indicating that it misses more attack samples. Overall, these results show that TCCA provides a better balance between attack detection and false alarms under temporal distribution shifts, although improving recall remains an important challenge.

#### 4.6.5. Robustness to Noise and Input Corruption

To assess the robustness of TCCA to input corruption, we conducted two tests: adding Gaussian noise to standardized features and randomly masking feature values by setting them to zero (the training-set mean after standardization). All tests used the same model checkpoint trained with seed 42. For each corruption level, five independent realizations were evaluated without retraining. Table 18 reports the mean and sample standard deviation across these realizations.

As shown in Table 18, TCCA maintains high detection performance under moderate Gaussian noise. Macro-F1 reaches 99.45% at σ=0.10 and remains at 97.69% at σ=0.20. Random masking has a greater impact. Macro-F1 decreases to 98.23% at 10% masking, 94.63% at 20%, and 90.45% at 30%. These results show that TCCA tolerates moderate input corruption, but its performance declines as more feature information is lost.

## 5. Discussion

The experimental results on CICIDS-2017 and CIC IIoT 2025 show that TCCA achieves strong detection performance, with mean accuracies of 99.89±0.05% and 99.81±0.13% across five random seeds, respectively. TCCA also outperforms CNN-LSTM and LCT-IDS in both accuracy and macro-F1 on the two datasets. These results suggest that combining spatial and temporal features through inter-layer cross-attention is more effective than relying on separate feature extraction or recurrent temporal modeling. The ablation results further support the value of inter-layer cross-attention. Compared with direct feature fusion in CNN–TCN, TCCA produces more distinct channel responses in the feature-map visualizations. The two unidirectional variants show different results across datasets. TCCA-S→T performs better on CICIDS-2017, while TCCA-T→S performs better on CIC IIoT 2025. This suggests that the contribution of each attention direction depends on the traffic characteristics. Full TCCA achieves the highest macro-F1 on both datasets, reaching 94.49±0.94% and 99.62±0.13%, respectively. These findings indicate that bidirectional cross-attention provides more effective feature integration than either unidirectional variant alone.

The efficiency results also show the practical value of the proposed architecture. The sequence-length analysis identifies L=32 as a suitable balance between detection performance and computational cost. Shorter sequences may miss useful temporal information, while longer sequences increase latency without further improving detection performance. TCCA uses parallel dilated temporal convolutions instead of recurrent layers, allowing temporal features to be processed in parallel. Under the evaluated workload, it achieves an average inference latency of 0.0034 ms/flow and a throughput of 297033.55 flows/s. Although cross-attention introduces additional computation, the model maintains a favorable balance between detection performance and inference efficiency. Despite these results, several limitations remain. First, detecting minority-class attacks is still challenging. For example, the Web Attack class has relatively few training samples, which may not fully capture its diverse traffic patterns. Second, SMOTE is applied to individual training records before sequence construction. Although this prevents validation and test data from entering the training process, the synthetic records do not have original temporal positions. Their use may therefore affect the temporal relationships within training sequences. Sequence-aware augmentation methods could help address this limitation while preserving temporal information.

Finally, the efficiency experiments were conducted on a GPU-based platform rather than resource-constrained edge devices. The reported results therefore do not establish performance on embedded hardware. Future work will further evaluate generalization under temporal distribution shifts and unseen attacks. It will also compare TCCA with additional recent TCN-, Transformer-, attention-, and edge-oriented IDS methods under the same experimental settings. In addition, hardware-in-the-loop experiments on platforms such as NVIDIA Jetson will be conducted to measure end-to-end latency, throughput, memory usage, and energy consumption under realistic industrial traffic.

## 6. Conclusions

This paper proposed TCCA, an inter-layer spatiotemporal cross-attention network for real-time IIoT intrusion detection. By integrating bidirectional cross-attention modules between the intermediate layers of a parallel CNN–TCN backbone, TCCA enables continuous mutual guidance between spatial and temporal representations while avoiding the recurrent latency bottlenecks of traditional CNN-RNN hybrid frameworks. Experimental results demonstrate that TCCA achieves multiclass classification accuracies of 99.89% on CICIDS-2017 and 99.81% on CIC IIoT 2025, while maintaining strong detection performance for difficult-to-detect web attacks. Ablation and benchmarking studies further show that inter-layer cross-attention is more effective than conventional late-fusion strategies, while TCCA achieves substantially lower inference latency and higher throughput than serial CNN-LSTM baselines under the evaluated GPU workload. Future work will focus on deploying TCCA on physical IIoT edge devices to evaluate real-world hardware performance, including latency, memory footprint, and power consumption. Cross-dataset evaluations will also be conducted to assess the model’s generalization capability against unseen attacks in heterogeneous industrial environments.

## Figures and Tables

**Table 1:** Benefits and limitations of representative IDS methods.

Model	Benefits	Limitations
TCN-based IDS [22]	Parallelizable temporal feature learning.	No explicit cross-branch feature interaction.
CNN-LSTM [16]	Joint spatial and temporal modeling with attention.	Sequential recurrent computation.
BiGAT-ID [30]	Complementary recurrent feature learning with attention.	Recurrent computation and late-stage feature fusion.
LCT-IDS [29]	Compact local and global feature modeling.	No explicit inter-flow temporal modeling.
ARN-based IDS [31]	Temporal modeling through hidden-state and input association.	Sequential state updates.
TCCA (ours)	Intermediate bidirectional cross-attention between parallel CNN and TCN branches.	Added attention overhead; physical edge deployment remains unverified.

**Table 2:** Class distribution of the processed CICIDS-2017 dataset.

Traffic Class	Number of Samples	Proportion
BENIGN	2,271,320	80.3203%
DoS	379,737	13.4286%
Port Scan	158,804	5.6158%
Patator	13,832	0.4891%
Web Attack	2180	0.0771%
Bot	1956	0.0692%

**Table 3:** Class distribution of the CIC IIoT 2025 dataset.

Traffic Class	Number of Samples	Proportion
BENIGN	400,672	58.44%
RECON	105,848	15.44%
DoS	57,736	8.42%
DDoS	56,692	8.27%
MITM	25,490	3.72%
MALWARE	24,177	3.53%
WEB	9040	1.32%
BRUTEFORCE	6016	0.88%

**Table 4:** Architecture specifications of the proposed TCCA model.

Module	Core Configuration	Output Shape
Input	68 selected features; sequence length 32	B×32×68
TCN stage 1	32 channels; kernel size 2; dilation 1; two-convolution residual block	B×32×32
CNN stage 1	Conv2D (1→64→32); BN, ReLU, and 2×2 max pooling	B×32×34×16
Cross-attention 1	Single-head bidirectional cross-attention; embedding dimension 32	Enhanced features
TCN stage 2	64 channels; kernel size 2; dilation 2; two-convolution residual block	B×64×32
CNN stage 2	Conv2D (32→64→64); BN, ReLU, and 2×2 max pooling	B×64×17×8
Cross-attention 2	Single-head bidirectional cross-attention; embedding dimension 64	Enhanced features
Feature fusion	Spatial projection (136→32) and element-wise addition	B×64×32
Classifier	Shared time-wise linear layer (64→C)	B×32×C

**Table 5:** Baseline hyperparameter search spaces and selected configurations.

Model	Search Space	Selected Configuration
CNN	Kernel: {3,5,7,9} $c_{1}$: {64,96,128,160} $c_{2}$: {32,48,60,64,80} $c_{3}$: {16,24,30,32,40}	LR: $9.33\times 10^{-4}$ WD: $5.83\times 10^{-4}$; *d*: 0.060 Kernel: 5 $\left(c_{1},c_{2},c_{3}\right)$: (160,60,40)
CNN-GRU	Kernel: {3,5,7,9} CNN channels: {32,64,96,128} GRU hidden size: {32,64,96,128} GRU layers: {1,2,3}	LR: $1.59\times 10^{-3}$ WD: $1.38\times 10^{-6}$ Kernel: 7; CNN channels: 32 GRU hidden size: 128; layers: 3
CNN-LSTM	Kernel: {3,5,7,9} Filters: {32,64,96,128} $h_{1}$: {32,64,96,128} $h_{2}$: {64,96,128} $p_{1}$: {2,5,10}; $p_{2}$: {2,5}	LR: $1.94\times 10^{-3}$ $\left(d_{1},d_{2}\right)$: (0.338,0.464) Kernel: 9; filters: 96 $\left(h_{1},h_{2}\right)$: (64,128) $\left(p_{1},p_{2}\right)$: (2,5)
LCT-IDS	Token dimension: {8,16,24,32} Heads: {1,2,4,8} Transformer layers: {1,2,3} MLP ratio: {2,3,4}	LR: $1.95\times 10^{-3}$ WD: $2.07\times 10^{-4}$; *d*: 0.100 Token dimension: 24 Heads: 2; Transformer layers: 3 MLP ratio: 2

Note: LR = learning rate; WD = weight decay; *d* = dropout rate; *c_i_* = channel width; *h_i_* = LSTM units; *p_i_* = pooling size.

**Table 6:** Accuracy and macro-averaged metrics on CICIDS-2017 (mean ± sample SD over five seeds). *p*-values are from two-sided paired *t*-tests of macro-F1 against TCCA.

Model	Accuracy (%)	Macro-P (%)	Macro-R (%)	Macro-F1 (%)	*p*-Value
CNN	98.52±0.37	86.47±4.02	90.72±4.52	87.55±3.16	0.0040
CNN-GRU	99.03±0.17	91.83±1.88	87.32±4.77	87.07±3.52	0.0081
CNN-LSTM	99.16±0.21	92.57±1.60	85.33±2.76	85.76±3.53	0.0059
LCT-IDS	99.33±0.56	92.68±6.66	85.73±3.50	84.67±1.40	0.0003
TCCA	99.89±0.05	94.46±1.95	94.97±0.53	94.49±0.94	–

**Table 7:** Accuracy and macro-averaged metrics on CIC IIoT 2025 (mean ± sample SD over five seeds). *p*-values are from two-sided paired *t*-tests of macro-F1 against TCCA.

Model	Accuracy (%)	Macro-P (%)	Macro-R (%)	Macro-F1 (%)	*p*-Value
CNN	95.11±0.17	97.05±0.47	90.55±0.33	93.57±0.40	<0.001
CNN-GRU	95.71±0.15	97.18±0.10	91.92±0.30	94.41±0.20	<0.001
CNN-LSTM	94.79±0.17	96.54±0.20	90.09±0.31	93.08±0.23	<0.001
LCT-IDS	95.06±0.19	96.31±0.63	90.39±0.53	93.16±0.55	<0.001
TCCA	99.81±0.13	99.70±0.08	99.54±0.23	99.62±0.13	–

**Table 8:** Class-specific performance of TCCA on CICIDS-2017.

Traffic Class	Precision (%)	Recall (%)	F1-Score (%)
BENIGN	99.96	99.94	99.95
Bot	88.73	96.68	92.53
DoS	99.85	99.96	99.91
Patator	99.24	99.53	99.39
Port Scan	99.81	99.97	99.89
Web Attack	86.28	75.00	80.25

**Table 9:** Class-specific performance of TCCA on CIC IIoT 2025.

Traffic Class	Precision (%)	Recall (%)	F1-Score (%)
BENIGN	99.91	99.95	99.93
BRUTEFORCE	99.16	98.17	98.66
DDoS	98.74	99.92	99.33
DoS	99.92	98.76	99.34
MALWARE	99.92	99.83	99.88
MITM	99.47	99.65	99.56
RECON	99.83	99.69	99.76
WEB	99.78	99.72	99.75

**Table 10:** Class-specific F1-score (%) comparison on CICIDS-2017.

Traffic Class	CNN	CNN-GRU	CNN-LSTM	LCT-IDS	TCCA
BENIGN	99.20	99.41	99.71	99.83	99.95
Bot	87.17	92.85	86.78	93.06	92.53
DoS	98.61	99.21	98.70	99.37	99.91
Patator	99.05	99.58	97.03	98.92	99.39
Port Scan	93.30	94.40	99.54	99.79	99.89
Web Attack	56.70	47.40	70.35	22.18	80.25

**Table 11:** Class-specific F1-score (%) comparison on CIC IIoT 2025.

Traffic Class	CNN	CNN-GRU	CNN-LSTM	LCT-IDS	TCCA
BENIGN	96.42	96.83	96.32	96.48	99.93
BRUTEFORCE	86.23	89.33	87.20	87.73	98.66
DDoS	93.18	95.06	93.42	94.29	99.33
DoS	95.47	97.05	95.55	96.38	99.34
MALWARE	94.73	96.36	95.63	96.06	99.88
MITM	93.29	95.49	93.35	94.19	99.56
RECON	89.84	91.59	89.73	90.22	99.76
WEB	93.52	94.50	93.45	93.60	99.75

**Table 12:** Joint ablation results on CICIDS-2017 and CIC IIoT 2025. All metrics are expressed as percentages.

Variant	CICIDS-2017			CIC IIoT 2025		
	Macro-P	Macro-R	Macro-F1	Macro-P	Macro-R	Macro-F1
CNN–TCN	91.61±0.98	87.77±1.22	89.34±0.38	99.45±0.12	99.20±0.07	99.32±0.06
TCCA-S→T	93.32±1.02	90.76±1.39	91.75±0.70	99.36±0.33	99.26±0.10	99.30±0.19
TCCA-T→S	93.55±1.55	89.18±1.74	91.02±1.51	99.52±0.18	99.49±0.08	99.50±0.11
First-layer fusion only	93.67±1.91	90.03±1.22	91.63±0.90	99.44±0.16	99.51±0.13	99.47±0.12
Last-layer fusion only	92.91±0.77	92.27±0.99	92.47±0.28	99.54±0.15	99.45±0.14	99.50±0.12
TCCA w/o SMOTE	94.92±0.84	92.75±0.75	93.74±0.57	99.57±0.13	99.55±0.06	99.56±0.06
**Full TCCA**	94.46±1.95	94.97±0.53	94.49±0.94	99.70±0.08	99.54±0.23	99.62±0.13

Note: Bold values indicate the best results in each column; the bold model name denotes our proposed model.

**Table 13:** Quantitative representation analysis on the held-out CIC IIoT 2025 test set. Results are reported as mean ± standard deviation.

Metric	CNN–TCN	TCCA	Δ	95% CI	*p*-Value
Channel redundancy					
Mean absolute channel correlation	0.2411±0.0211	0.2349±0.0299	−0.0062	[−0.0071,−0.0052]	<0.001
Signed channel correlation	0.1303±0.0309	0.0735±0.0207	−0.0567	[−0.0580,−0.0555]	<0.001
Normalized effective rank	0.2397±0.0252	0.2582±0.0346	+0.0184	[0.0175,0.0194]	<0.001
Class separability					
Fisher ratio	1.7506±0.2103	3.6169±0.0690	+1.8663	[1.3976,2.1727]	<0.004
Silhouette score	0.4040±0.0104	0.4794±0.0087	+0.0754	[0.0686,0.0972]	<0.004
Davies–Bouldin index	0.9296±0.0145	0.7614±0.0110	−0.1682	[−0.1863,−0.1478]	<0.004
Linear-probe Macro-F1	0.9884±0.0029	0.9918±0.0021	+0.0034	[−0.0026,0.0110]	0.244

**Table 14:** Computational complexity and detection efficiency on CIC IIoT 2025.

Model	Params (M)	Memory (MiB)	FLOPs (G)	E2E (ms/flow)	Inference (ms/flow)	Throughput (flows/s)
CNN	0.0636	114.89	21.01	0.0026	0.0023	438,648.16
CNN-GRU	0.7596	967.80	103.94	0.0273	0.0269	37,106.21
CNN-LSTM	0.2190	155.18	12.59	0.0225	0.0220	45,539.07
LCT-IDS	0.0188	96.09	3.37	0.0097	0.0093	107,544.16
TCCA	0.1454	47.32	2.03	0.0037	0.0034	297,033.55

**Table 15:** Sensitivity of TCCA to sequence length.

*L*	Accuracy (%)	Macro-F1 (%)	Sequence Latency (ms)
8	99.23	97.97	0.13
16	99.73	99.00	0.13
32	99.78	99.53	0.14
64	99.58	99.42	0.21

**Table 16:** Stability of TCCA across five random seeds.

Dataset	Accuracy (%, Mean ± SD)	Macro-F1 (%, Mean ± SD)
CICIDS-2017	99.89 ± 0.05	94.49 ± 0.94
CIC IIoT 2025	99.81 ± 0.13	99.62 ± 0.13

**Table 17:** Temporal generalization performance on CIC IIoT 2025. All values are expressed as percentages.

Metric	CNN-GRU	LCT-IDS	TCCA
Accuracy	83.44	85.79	**92.00**
Macro-F1	83.37	85.68	**91.66**
Attack Precision	76.02	79.51	**97.10**
Attack Recall	89.62	**90.04**	83.83
Attack F1	82.27	84.45	**89.98**
False-positive rate	21.20	17.40	**1.88**
AUROC	93.27	88.74	**95.67**
AUPRC	93.69	80.57	**96.44**

Note: Bold values indicate the best performance for each metric.

**Table 18:** Noise sensitivity and corrupt-input robustness of TCCA on CIC IIoT 2025. Results are reported as mean ± sample standard deviation over five independent corruption realizations.

Input Condition	Level	Accuracy (%)	Macro-P (%)	Macro-R (%)	Macro-F1 (%)
Clean	–	99.87	99.75	99.53	99.64
Gaussian noise	σ=0.01	99.86±0.00	99.75±0.00	99.52±0.00	99.63±0.00
Gaussian noise	σ=0.05	99.84±0.02	99.73±0.02	99.49±0.10	99.61±0.06
Gaussian noise	σ=0.10	99.61±0.07	99.54±0.05	99.36±0.14	99.45±0.09
Gaussian noise	σ=0.20	97.54±0.15	97.84±0.13	97.68±0.48	97.69±0.30
Random masking	5%	98.90±0.17	98.96±0.13	99.14±0.13	99.04±0.09
Random masking	10%	97.84±0.08	98.04±0.15	98.51±0.35	98.23±0.25
Random masking	20%	95.30±0.33	95.71±0.29	94.30±0.45	94.63±0.41
Random masking	30%	92.98±0.28	93.43±0.26	89.40±0.41	90.45±0.32

## Data Availability

The raw datasets used in this study are publicly available. The CICIDS-2017 dataset is available from the Canadian Institute for Cybersecurity at [https://www.unb.ca/cic/datasets/ids-2017.html (accessed on 20 February 2026)]. The CIC IIoT 2025 dataset is available at [https://www.unb.ca/cic/datasets/iiot-dataset-2025.html (accessed on 25 March 2026)].

## Supplementary Materials

The following supporting information can be downloaded at https://www.mdpi.com/article/10.3390/s26206648/s1: Table S1. CICIDS-2017; Table S2. CIC-IIoT-2025.

## References

[1] Zhang Z., Zeng L., Zhu D., Tan H., Wang L., Li Z., Gu Z. (2025). GBFKAN: An Adaptive Multilayer Interpretable Architecture for Intrusion Detection in Various Internet of Things Scenarios. IEEE Internet Things J..

[2] Zhu L., Fu X., Liu X., Du S. (2025). Modeling and analysis of cascade failures in Industrial Internet of Things based on task decomposition and service communities. Comput. Ind. Eng..

[3] Karami A., Gebremedhin M.A. (2026). Securing TTEthernet clock synchronization in the IIoT: A multilayered defense against intelligent cyber-attacks. Clust. Comput..

[4] Behnke I., Austad H. (2024). Real-Time Performance of Industrial IoT Communication Technologies: A Review. IEEE Internet Things J..

[5] Alageel A., Maffeis S. (2026). Investigation of Advanced Persistent Threats Network-Based Tactics, Techniques and Procedures. Comput. Netw..

[6] Shaukat S.U., Khan S., Parkinson S. (2025). A Review on Multi-Step Attack Detection. IEEE Access.

[7] Jamshidi S., Nafi K.W., Nikanjam A., Khomh F. (2025). Evaluating machine learning-driven intrusion detection systems in IoT: Performance and energy consumption. Comput. Ind. Eng..

[8] Al-Shurbaji T., Anbar M., Manickam S., Hasbullah I.H., Alfriehat N., Alabsi B.A., Alzighaibi A.R., Hashim H. (2025). Deep Learning-Based Intrusion Detection System for Detecting IoT Botnet Attacks: A Review. IEEE Access.

[9] Neha, Kajal A. (2025). A Comprehensive Literature Review: Feature Engineering Techniques in Enhancing Machine and Deep Learning Models Optimization for Intrusion Detection Systems. Proceedings of the 5th International Conference on Artificial Intelligence and Smart Energy (ICAIS 2025).

[10] Zhao P., Wang H., Liu Y., Zhang S., Zhao X., Chi Z. (2026). Review of Deep Learning in Network Intrusion Detection. Comput. Eng. Appl..

[11] Anand M., Muthurajkumar S. (2025). An intelligent IDS using bagging based fuzzy CNN for secured communication in vehicular networks. Sci. Rep..

[12] de Oliveira J.A., dos Santos A.F.P., Salles R.M. (2025). RNN for intrusion detection in digital substations based on the IEC 61850. J. Inf. Secur. Appl..

[13] Dash N., Chakravarty S., Rath A.K., Giri N.C., AboRas K.M., Gowtham N. (2025). An optimized LSTM-based deep learning model for anomaly network intrusion detection. Sci. Rep..

[14] Murugesan S., Ramkumar N., Usharani P., Ramalingam S., Elavarasi R., Priya S. (2025). Enhancing IoT and Edge Computing Security with PCA-Driven Dimensionality Reduction and TCN-based Intrusion Detection. Proceedings of the 2025 6th International Conference on Smart Electronics and Communication (ICOSEC).

[15] Bamber S.S., Katkuri A.V.R., Sharma S., Angurala M. (2025). A hybrid CNN-LSTM approach for intelligent cyber intrusion detection system. Comput. Secur..

[16] Alashjaee A.M. (2025). Deep learning for network security: An Attention-CNN-LSTM model for accurate intrusion detection. Sci. Rep..

[17] Huang W. (2025). Research on Multi Modal Network Intrusion Detection Method Based on Deep Neural Network. Proceedings of the 2025 IEEE 8th International Conference on Information Systems and Computer Aided Education (ICISCAE).

[18] Alsarhan A., Alauthman M., Alshdaifat E., Al-Ghuwairi A.R., Al-Dubai A. (2023). Machine Learning-driven optimization for SVM-based intrusion detection system in vehicular ad hoc networks. J. Ambient Intell. Humaniz. Comput..

[19] Pramilarani K., Vasanthi Kumari P. (2024). Cost based Random Forest Classifier for Intrusion Detection System in Internet of Things. Appl. Soft Comput..

[20] Wang L.H., Dai Q., Du T., Chen L.F. (2024). Lightweight intrusion detection model based on CNN and knowledge distillation. Appl. Soft Comput..

[21] Mandela N., Etyang F. (2025). Comparative analysis of deep learning models for effective denial of service (DoS) attack detection in network security. J. Electr. Syst. Inf. Technol..

[22] Benfarhat I., Goh V.T., Siow C.L., Lee I.E., Sheraz M., Ngu E.E., Chuah T.C. (2025). Advanced Temporal Convolutional Network Framework for Intrusion Detection in Electric Vehicle Charging Stations. IEEE Open J. Veh. Technol..

[23] Duan X., Wang K., Fu Y., Liu T., Yu Y., Xu J., Wang L. (2025). Abnormal Traffic Detection Method Based on DCNN-GRU Architecture in SDN. Int. J. Intell. Syst..

[24] Alsuwat E., Alsuwat H. (2025). High-performance intrusion detection using hybrid BiLSTM–TCN model with adaptive sine–cosine algorithm for hyperparameter optimization. J. Supercomput..

[25] Ji C., Liu H., Dai W. (2025). Hybrid Model for Network Traffic Anomaly Detection Based on Parallel Two-Stage Feature Fusion. IEEE Access.

[26] Anjum N., Latif Z., Chen H. (2025). Security and privacy of industrial big data: Motivation, opportunities, and challenges. J. Netw. Comput. Appl..

[27] Khan F.U., Mian A.N. (2025). Low-latency and interpretable intrusion detection for IIoT using self-supervised learning with entropy-based masking. Comput. Electr. Eng..

[28] Nagini R.V.S.S.S., Prabhakar P.B.E., Balsem Z.A., Soni S., Mukuntharaj C., Hadi A.A.A.K., Alhayaly M.A. (2025). Lightweight Intrusion Detection Algorithm for Edge Computing in Industrial IoT Applications. Proceedings of the 2025 3rd International Conference on Cyber Resilience (ICCR).

[29] Tao X., Du Y., Jiang Q., Zhao W., Xu K., Meng X. (2026). LCT-IDS: A Lightweight Transformer-Based Architecture for IoT Intrusion Detection. IEEE Internet Things J..

[30] Gueriani A., Kheddar H., Mazari A.C., Ghanem M.C. (2026). A robust cross-domain IDS using BiGRU-LSTM-attention for medical and industrial IoT security. ICT Express.

[31] Liu Z., Ye D., Yang C., Ding Y., Liu Y., Tang L., Chen C. (2026). Take Attention as Gate: An Associative Recurrent Network-Based Intrusion Detection Method for Industrial Control Network. IEEE Trans. Netw. Serv. Manag..

[32] Lin Y., Wei Y., Chen D., Li Y., Erkan U., Toktas A., Gao S., Zhang Y. (2026). Cryptanalysis and Improvement of a Video Cryptosystem via Chaos and S-Box. ACM Trans. Multimed. Comput. Commun. Appl..

[33] Lee J., Chang J., Lee D., Choi J. (2026). CA^2^ST: Cross-Attention in Audio, Space, and Time for Holistic Video Recognition. IEEE Trans. Pattern Anal. Mach. Intell..

[34] Sharafaldin I., Habibi Lashkari A., Ghorbani A.A. (2019). A Detailed Analysis of the CICIDS2017 Data Set. Proceedings of the Information Systems Security and Privacy.

[35] Firouzi A., Dadkhah S., Maret S.A., Ghorbani A.A. (2025). Datasense: A real-time sensor-based benchmark dataset for attack analysis in iiot with multi-objective feature selection. Electronics.

[36] Tan J., Lu X., Zhang G., Yin C., Li Q. (2021). Equalization Loss v2: A New Gradient Balance Approach for Long-Tailed Object Detection. Proceedings of the IEEE/CVF Conference on Computer Vision and Pattern Recognition (CVPR).

[37] Jose J., Jose D.V. (2023). Deep learning algorithms for intrusion detection systems in internet of things using CIC-IDS 2017 dataset. Int. J. Electr. Comput. Eng..

[38] Mynuddin M., Khan S.U., Chowdhury Z.U., Islam F., Islam M.J., Hossain M.I., Ahad D.M.A. (2024). Automatic network intrusion detection system using machine learning and deep learning. Proceedings of the 2024 IEEE International Conference on Artificial Intelligence and Mechatronics Systems (AIMS).

[39] Appathurai A., Bachan P., Kaushik R., Tamizhselvi A., Jodha R., Singh K. (2026). Intrusion detection system with Walrus optimization algorithm (WOA) and BiGRU-CNN for securing IoT systems. Int. J. Inf. Technol..

