# Supplementary material for: TCCA: An Inter-Layer Spatiotemporal Cross-Attention Network for IIoT Attack Traffic Detection
Source: Sensors (Basel). 2026 Oct 21;26(20):6648. doi: 10.3390/s26206648 (PMC13709088; doi:10.3390/s26206648)
Supplement: Supplementary file 1 [file sensors-26-06648-s001.zip › sensors-4601533-supplementary.pdf]

Table S1. CICIDS-2017

No.	Feature	No.	Feature
1	Protocol_0	35	Bwd Header Length
2	Protocol_6	36	Fwd Packets/s
3	Protocol_17	37	Bwd Packets/s
4	Flow Duration	38	Min Packet Length
5	Total Fwd Packets	39	Max Packet Length
6	Total Backward Packets	40	Packet Length Mean
7	Total Length of Fwd Packets	41	Packet Length Std
8	Total Length of Bwd Packets	42	Packet Length Variance
9	Fwd Packet Length Max	43	FIN Flag Count
10	Fwd Packet Length Min	44	SYN Flag Count
11	Fwd Packet Length Mean	45	PSH Flag Count
12	Fwd Packet Length Std	46	ACK Flag Count
13	Bwd Packet Length Max	47	URG Flag Count
14	Bwd Packet Length Min	48	Down/Up Ratio
15	Bwd Packet Length Mean	49	Average Packet Size
16	Bwd Packet Length Std	50	Avg Fwd Segment Size
17	Flow Bytes/s	51	Avg Bwd Segment Size
18	Flow Packets/s	52	Fwd Header Length.1
19	Flow IAT Mean	53	Subflow Fwd Packets
20	Flow IAT Std	54	Subflow Fwd Bytes
21	Flow IAT Max	55	Subflow Bwd Packets
22	Flow IAT Min	56	Subflow Bwd Bytes
23	Fwd IAT Total	57	Init_Win_bytes_forward
24	Fwd IAT Mean	58	Init_Win_bytes_backward
25	Fwd IAT Std	59	act_data_pkt_fwd
26	Fwd IAT Max	60	min_seg_size_forward
27	Fwd IAT Min	61	Active Mean
28	Bwd IAT Total	62	Active Std
29	Bwd IAT Mean	63	Active Max
30	Bwd IAT Std	64	Active Min
31	Bwd IAT Max	65	Idle Mean
32	Bwd IAT Min	66	Idle Std
33	Fwd PSH Flags	67	Idle Max
34	Fwd Header Length	68	Idle Min

Table S2. CIC-IIoT-2025

No.	Feature	No.	Feature
1	log_data-ranges_avg	35	network_packets_dst_count
2	log_data-ranges_max	36	network_packets_src_count
3	log_data-ranges_min	37	network_payload-length_avg
4	log_data-ranges_std_deviation	38	network_payload-length_max
5	log_data-types_count	39	network_payload-length_min
6	log_interval-messages	40	network_payload-length_std_deviation
7	log_messages_count	41	network_ports_all_count
8	network_fragmentation-score	42	network_ports_dst_count
9	network_fragmented-packets	43	network_ports_src_count
10	network_header-length_avg	44	network_protocols_all_count
11	network_header-length_max	45	network_protocols_dst_count
12	network_header-length_min	46	network_protocols_src_count
13	network_header-length_std_deviation	47	network_tcp-flags-ack_count
14	network_interval-packets	48	network_tcp-flags-fin_count
15	network_ip-flags_avg	49	network_tcp-flags-psh_count
16	network_ip-flags_max	50	network_tcp-flags-rst_count
17	network_ip-flags_min	51	network_tcp-flags-syn_count
18	network_ip-flags_std_deviation	52	network_tcp-flags-urg_count
19	network_ip-length_avg	53	network_tcp-flags_avg
20	network_ip-length_max	54	network_tcp-flags_max
21	network_ip-length_min	55	network_tcp-flags_min
22	network_ip-length_std_deviation	56	network_tcp-flags_std_deviation
23	network_ips_all_count	57	network_time-delta_avg
24	network_ips_dst_count	58	network_time-delta_max
25	network_ips_src_count	59	network_time-delta_min
26	network_mss_avg	60	network_time-delta_std_deviation
27	network_mss_max	61	network_ttl_avg
28	network_mss_min	62	network_ttl_max
29	network_mss_std_deviation	63	network_ttl_min
30	network_packet-size_avg	64	network_ttl_std_deviation
31	network_packet-size_max	65	network_window-size_avg
32	network_packet-size_min	66	network_window-size_max
33	network_packet-size_std_deviation	67	network_window-size_min
34	network_packets_all_count	68	network_window-size_std_deviation
